# De- and Re-Structuring of Starch to Control the Melt and Solid State Visco-Elasticity as Method for Getting New Multi Component Compounds with Scalable Properties

**DOI:** 10.3390/polym16213063

**Published:** 2024-10-30

**Authors:** Doina Dimonie, Ramona-Marina Grigorescu, Bogdan Trică, Monica Raduly, Celina-Maria Damian, Roxana Trusca, Alina-Elena Mustatea, Stefan-Ovidiu Dima, Florin Oancea

**Affiliations:** 1 Chemical Engineering and Biotechnologies Faculty’ Doctoral School, National University of Science and Technology Politehnica Bucharest, 313 Splaiul Independentei, 060042 Bucharest, Romania; ddimonie@yahoo.com (D.D.); alinaelenamustatea84@yahoo.com (A.-E.M.); 2 National Institute for Research and Development in Chemistry and Petrochemistry, 202 Splaiul Independentei Street, 060021 Bucharest, Romania; ramona.grigorescu@icechim.ro (R.-M.G.); radulymonica@yahoo.com (M.R.); ovidiu.dima@icechim.ro (S.-O.D.); florin.oancea@icechim.ro (F.O.); 3 National Centre for Micro and Nanomaterials and National Centre for Food Safety, National University of Science and Technology Politehnica Bucharest, 060042 Bucharest, Romania; celina.damian@yahoo.com (C.-M.D.); roxanatrusca@yahoo.com (R.T.); 4 Advanced Polymer Materials Group, National University of Science and Technology Politehnica Bucharest, Gh. Polizu Street, No. 1-7, 010061 Bucharest, Romania

**Keywords:** starch, PVA, entanglement, melt and solid-state viscoelastic properties, thermodynamically stability

## Abstract

The aim of the article was to design and develop new thermodynamically stable starch-based compounds, with scalable properties, that are melt-processable into finished products by classic or 3D printing methods. This is based on phenomena of de-structuring, entanglement compatibilization, and re-structuring of starch, along with the modification of the polymer, polyvinyl alcohol (PVA), by following an experimental sequence involving pre-treatment and melt compounding in two stages. The new compounds selection was made considering the dependence of viscoelastic properties on formulation and flowing conditions in both the melted and solid states. Starting from starch with 125 °C glass transition and PVA with a T_g_ at 85 °C, and following the mentioned experimental sequence, new starch-PVA compounds with a high macromolecular miscibility and proven thermodynamic stability for at least 10 years, with glass transitions ranging from −10 °C to 50 °C, optimal processability through both classical melt procedures (extrusion, injection) and 3D printing, as well as good scalability properties, were achieved. The results are connected to the approaches considering the relationship between miscibility and the lifetime of compounds with renewable-based polymer content. By deepening the understanding of the thermodynamic stability features characterizing these compounds, it can be possible to open the way for starch usage in medium-life compositions, not only for short-life applications, as until now.

## 1. Introduction

Polymeric materials, obtained by melt compounding, are multicomponent and/or multiphase systems obtained by structuring techniques [1,2,3,4], which represent the process of arranging the material components in an order manner to achieved the highest possible level of functional properties [5,6]. The structuring is performed through methods that control the mobility of the macromolecular segments, the reactivity of the components, the morphology at the interfaces, and/or the melt flow properties [7,8,9,10,11]. In contrast to thermodynamically unstable polymer systems that can demix, sometimes immediately after being formed (and thus are not suitable for scaling), well-executed structuring processes lead to thermodynamically stable compounds with proper lifetime [12,13,14,15,16].

Starch is a semi-crystalline composite [17], of scientific and applicative interest, whose functional properties can be modified by chemical and/or physical methods [18,19], many of which are scalable [20]. It can be physically modified through melt compounding with polymers of renewable (biodegradable polyesters [21]), or non-renewable origin (polyvinyl alcohol (PVA) [20,22,23]), reinforcements (layered silicates [11,24,25,26]), fillers, plasticizers, flow agents, and other additives. Starch gains elasticity if it is modified with PVA [27], a polymer that, due to its water solubility, makes the compound completely water disintegrable, aligning with the circular economy principle of minimal environmental impact [20]. Starch–PVA blends have been intensively studied for variations in functional properties with composition, the influence of humidity, compounding conditions [19,28,29,30,31,32], stiffness control via PVA structure [33], plasticizers leaching [34], antibacterial properties [35,36], and the formation of multilayer structures [37]. For high-tonnage applications, these compounds are mainly designed as multi-component systems, with each component (and the specific interactions between them) playing an explicit role in reaching the desired functional properties [38,39,40,41,42,43], along with the characteristics of the melt compounding facilities (extruder, die, takeoff device, etc.) and the operation conditions (temperature, pressure, shear rate, etc.) [44,45,46,47,48].

The biggest difficulty in achieving the PVA–starch compounds using melt compounding techniques arises because the two polymers have an extremely high hydroxyl groups content and well-defined semi-crystalline morphologies, and amylopectin, one of the starch components, has a branched structure [49]. For this reason, both starch and PVA degrade before melting [50,51], making it challenging to de-structure them so that their macromolecules to flow freely in the melted state, allowing for a new re-structuring process as result of the new attractions between the starch macromolecules and those of PVA.

The miscibility of polymers is defined as the formation of stable, homogeneous blends that exhibit the macroscopic properties expected of a single-phase material [52,53,54] and can be studied via classical methods (DSC, FTIR, etc.) or current methods such as Molecular Dynamics Simulation [55]. FTIR provides information about the types of interactions between the functional groups of the blend components [56,57,58], which helps to establish correlations between compatibility, miscibility, and functional properties [59] (e.g., mechanical [60], thermal [61], solubility [62,63]). The compatibility analysis of the two polymers has been conducted [64], but not in correlation with the intrinsic physical transformations of the interactions between the two polymers’ macromolecules or by using the viscoelastic properties in the selection of the processing technique for the finished product.

The purpose of the article was to design and carry out a melt compounding procedure for the de-structuring of starch and PVA and their re-structuring into new multicomponent blends with advanced miscibility and scalable properties, and to utilize the viscoelastic properties in the melted or solid state to select formulations and processing techniques.

## 2. Materials and Methods

### 2.1. Designing of New Compounds, Procedure and Materials

In the first stage, the de-structuring of the starch and PVA was achieved by canceling the intermolecular attractions responsible for the semi-crystalline morphologies of these two polymers and allowing the macromolecules to reach a state of free melt flow. For this purpose, the solid polymers were pre-compounded with the liquid plasticizer under controlled thermo-mechanical conditions so that, in the end, following the adsorption of the plasticizer on the two polymer particle surface, the resulting powder flowed freely as if it did not contain absorbed liquid. After this stage, melt compounding followed under certain conditions of temperature and shear rate to enable the melted macromolecules entanglement (entanglement compatibilization) during the compounding process and the formation of new physical bonds of 1–3 kcal·mol^−1^, similar to hydrogen bonds [65,66], this time between the starch macromolecules and those of PVA (re-structuring). In order to ensure a wide range of glass transition values, so that the resulting compounds would have optimal functional properties for various applications through subsequent additional modification, a high level of plasticization was used. The level of miscibility (de-structuring, entanglement compatibilization, and re-structuring) was studied using FTIR and SEM. In the conducted experiments, the independent variables were the formulation and flow conditions, while the dependent variables were the melt’s viscous properties (shearing stress, shear rate, fluidity, flow resistance), the melt elastic properties (extrudate swelling at the nozzle exit), and the solid-state viscoelastic properties (dynamo-mechanical behavior). The studied melt rheological properties characterize the melt processing into the finished product by techniques of practical interest: extrusion [67,68,69,70,71,72,73,74,75], injection [76,77,78,79,80,81], and 3D printing [9]. Depending on the relationships between viscoelastic properties, formulations, and flow conditions, compositions suitable for melt processing into finished products, for physic-mechanical characterization, and for verifying their scalability were selected.

The compounding was performing using a usual Brabender procedure under chosen conditions (90–170 °C temperature, 3–15 min compounding time, 75 rpm–150 rot/min rotors speed). After melt compounding, each blend was rolled into sheets using a laboratory roller under selected conditions (40–80 °C temperature and 21 rpm/30 rpm roll speed). The laboratory-selected formulations were compounded and granulated using a double-screw extruder (150–175 °C, 25 rpm) and were then shaped into filaments on a Gottfert rheometer (95–125 °C), which were subsequently granulated. The obtained granules were further extruded/injected/3D printed. The filaments for 3D printing that required distinct sizes were obtained on a single-screw extruder (95–125 °C, 5–75 rpm) equipped with a line for calibration, cooling, pulling, and rolling of the resulted filaments. The injection tests were conducted using an injection device (Ray-Ran injection device) under chosen conditions (110–170 °C-pressing temperature, 5 min–10 min–10 min as pre-heating, pressing and cooling time, 200 bar-pressing time). The 3D printing was performed using a 3D printer, model UP Plus 2.

The starch/PVA blending ratio ranged from 0–100/100–0. Experiments were conducted with (5–80) [p] plasticizer (glycerin) and (0–5) [p] of flow agent added to 100% blend of starch and PVA. Corn starch (Amulet 100/Asylum Romania) with 70% amylopectin and 125 °C glass transition was used. It loses up to 10.63% from its weight by 210 °C and almost 73.35% between 200 °C and 400 °C. Partially hydrolyzed polyvinyl alcohol (84% hydrolysis degree, Du Pont, Wilmington, DE, USA) with a T_g_ of 85 °C and a mass loss of approximately 3.01% up to 120 °C and 81.43% between 170 °C and 400 °C was used as a modifier for starch. Glycerin (Reagent Chemical Services Ltd., Hudson, UK, GLYC-2593-06) was used as a plasticizer, calcium stearate (technical grade, Parchem CAS1592-23-0, New Rochelle, NY, USA) as a flow agent, and a combination of 3/1 sterically hindered phenol and sterically hindered phosphite (BASF, Ludwigshafen an Rhein, Germany) as a thermal stabilizer.

**Figure 1:**
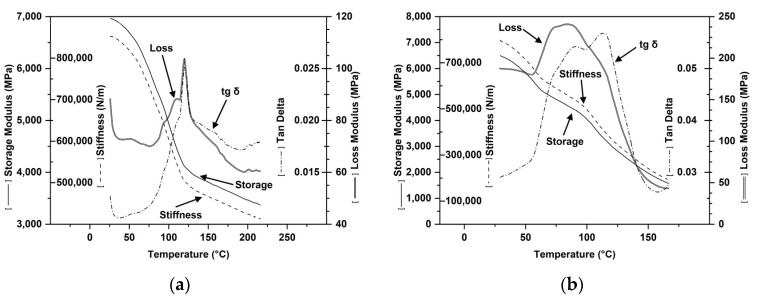
The thermal behavior of starch (**a**) and PVA (**b**) (DMA registrations).

The thermal behavior of the starch and PVA used is presented in Figure 1. The starch has a glassy plateau area up to approx. 40 °C, followed by a wide glass transition with a maximum at 125 °C, and then, starting from 180 °C, another plateau indicating the highly elastic state (Figure 1a). For PVA, the glass plateau ends at approx. 50 °C, the glass transition is recorded at 85 °C, and the highly elastic zone begins at approx. 150 °C (Figure 1b).

### 2.2. Characterization

#### 2.2.1. Miscibility

##### Chemical Structure

The FTIR spectra were recorded using a Jasco FTIR 6300 Spectrometer (Jasco Corporation, Tokyo, Japan) equipped with a Specac Golden Gate ATR (KRS5 lens), in the range of 400–4000 cm^−1^ (32 scans at 4 cm^−1^ resolution). Normalization was achieved by relating the intensity of each absorption to that of the highest one found at 1025 cm^−1^. The analyzed samples were dried for 4 h at 80 °C before recording. The assignments for the FTIR peak were conducted based on: [56] for PVA, [57,82,83,84,85] for starch, and [86] for glycerol.

##### Morphological Structure

Scanning electron microscopy (SEM) was performed using a Quanta INSPECT F scanning electron microscope -FEI-Philips, Netherlands, equipped with an electron field emission gun (EFG) with a resolution of 1.2 nm. Lyophilization was performed in a CHRIST ALPHA 1-2 LD plus freezer under the following conditions: main drying at −42.9 °C and 0.091 mbar, followed by finally drying at −43.2 °C and 0.012 mbar.

#### 2.2.2. Vasco-Elastic Behavior

##### In the Melted State

The Rheological Properties of the Melts

These were studied using the flow index method with a capillary rheometer, type DYNISCO 4000 LMI, Franklin, MA, USA, equipped with a nozzle with an L/R = 8 ratio (l = 8 mm, diameter = 2.09 mm). The measurements were performed in the temperature range of 125–155 °C, at load 2.16 kg, 3.8 kg, 5 kg, and 10 kg, cutting the extrudate at 20s. The following melt properties were measured: shear stress (SS—shear stress), shear rate (SR—shear rate), melt flow index (MFI—melt flow index), and melt flow resistance (DV—dynamic viscosity).

Extrudates Swelling

This was studied using a capillary rheometer (piston plastometer, Polireo—01) with two nozzles of lengths 8.2 mm and diameters of 1.27 mm and 2.272 mm. Measurements were made at weights of 5.1 kg, 7.1 kg, and 10.15 kg in the temperature range between 130 °C and 150 °C. The observation of the extrudate at the nozzle exit was performed using a scope with an attached comparator. The degree of swelling (β) was measured by relating the maximum diameter of the extrudate to that of the nozzle.

##### In the Solid State

Dynamic Mechanical Properties (DMA)

This was performed using a TRITEC 2000 B (Triton Technologies, Mansfield, MA, USA) in single cantilever mode, from −60 to 100 °C with 1Hz frequency and 5 °C/min heating rate.

Mechanical Properties

Testing was conducted using a Universal Testing Machine Instron 3382, with 100 kN load cell at room temperature, with prior temperature and humidity conditioning for 48 h. The specimens (5A type according to ISO 527 [87]) were tested for each sample and analyzed using Bluehill Software 8 at a rate of 5 mm/min until fragmentation occurred. The experiments were performed on parallel specimens to ensure consistency. Measurements were performed five times for each sample, and the average value was reported.

### 2.3. Other Properties

#### 2.3.1. Density

Density was determined following ASTM 792 [88].

#### 2.3.2. Hardness

Hardness was determined following ASTM 785-23 [89].

#### 2.3.3. Color

Visual estimates and comparison with a white color blend.

#### 2.3.4. Durability–Qualitative Assessments of Mechanical Properties over Almost 10 Years

This refers to the periodic qualitative estimates of fracture behavior. Tracking the loss of integrity of the items made from the selected compounds.

## 3. Results

### 3.1. Compounding

#### 3.1.1. Deficiencies at Preliminary Compounding of Starch with PVA (Figure 2)

**Figure 2:**
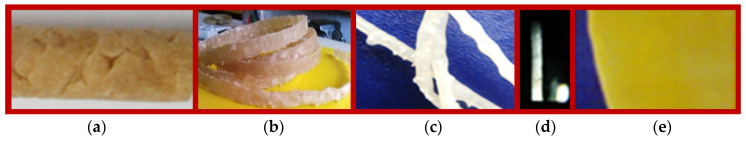
Deficiencies observed during the primary compounding of some starch–PVA blends (**a**) incomplete melting; (**b**) roughness; (**c**) gaseous inclusions, (**d**) extrudate swelling of the extrudate; (**e**) coloring [9].

Preliminary compounding tests showed many deficiencies, such as incomplete melting (Figure 2a), rough surfaces (Figure 2b), gaseous inclusions (Figure 2c), swelling of the extrudate (Figure 2d), colored items (Figure 2e), and plasticizers leaching (sometimes immediately after compounding).

#### Compounding Using Polymer as Powder with Similar Diameter (Figure 2)

It was observed that incomplete melting (Figure 2a,b) and rough surfaces (Figure 2b) no longer appear if both the melting and the melt flow are controlled by using the two polymers as solid particles with similar diameter [9,90]. If the particles have similar diameters, the two polymers melt at approximately the same time. Melting time will be longer for larger diameter particles. At the same compounding time, depending on its value, melted, un-melted, and/or degraded particles may result. For example, if the goal is to melt particles with larger diameters and the compounding time is increased, then the small particles may degrade. Conversely, if the time is adjusted according to the particles with small diameters and the compounding time is decreased, the large particles may be only partially melted. In the first case, the extrudate will have a rough appearance, and in the second case, it will contain un-melted particles on its surface.

#### Pre-Conditioning of the Solid and Liquid Components before Melt Compounding (Figure 3)

**Figure 3:**
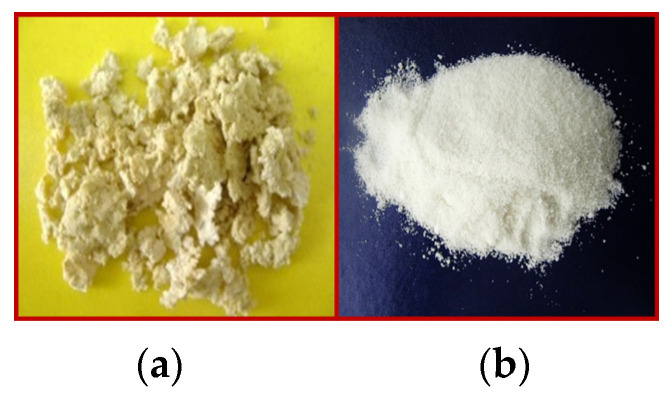
The appearance of the blend of starch and PVA solid powders with glycerin, without (**a**) or with (**b**) “dry-blend” pre-mixing before melt compounding.

The leaching of the plasticizer was eliminated by mixing the liquid components with the solid ones (Figure 3) in controlled thermo-mechanical conditions before melt compounding, using a procedure similar to [91]. If the mixture, consisting of the two solid polymers and the liquid plasticizer, is not pre-treated before melt compounding, it will have an agglomerated appearance (Figure 3a). However, if it has been subjected to this pre-treatment it will flow as a single powder that does not contain liquid (Figure 3b).

#### Drying of Polymers Before Melt Compounding

Based on previously obtained results [26], the strong hygroscopicity of the two polymers was revealed, and it was found that they are highly degradable between 140 °C and 220 °C. In the studies conducted, only dry polymers (4 h at 80 °C) were used. Additionally, formulations and processing conditions that enable the melt compounding below 140 °C were identified.

### 3.2. Miscibility of Binary Blends

#### 3.2.1. Chemical Structure (Figure 4, Table S1—In Supplementary Data)

**Figure 4:**
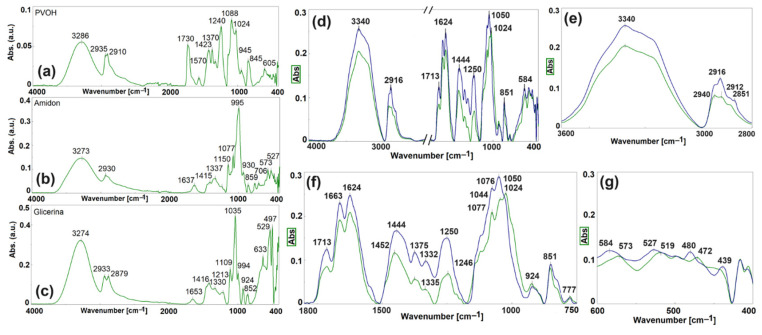
FTIR spectra of PVA (**a**), starch (**b**), glycerin (**c**), blends with 20% (blue curve) and 70% (green curve) starch over the entire spectral range (**d**), and in the selected spectral ranges: 3000 cm^−1^–2800 cm^−1^ (**e**), 1800 cm^−1^–750 cm^−1^ (**f**), and 600 cm^−1^–400 cm^−1^ (**g**).

The gross, appreciable, and small FTIR spectral changes describe the interactions between the functional groups of the polymers in the blends [56]. “Gross spectral changes” describe entirely new spectral features or changes in intensities greater than 50%. “Appreciable spectral changes” describe case where the same features are present in the spectra but have been shifted by more than one width at half height of the absorption or have changed in intensity by more than 30%. “Small spectral shifts” are those <10 cm^−1^ [24,82,92]. As shown in Figure 4 and Table S1, the new compounds, both with 20% and 70% starch, display distinct absorptions compared to the individual components across all spectral domains. If the three components of the blends (starch, PVA, glycerin) have a total of 40 absorption peaks, the two blends have only nine FTIR absorption peaks. The absorption maxima of these nine peaks occur at wavelengths completely different from those of the individual components, meaning that the described procedure of de-structuring, entanglement, compatibilization, and re-structuring has resulted in advanced miscibility of the studied blends.

#### 3.2.2. Miscibility—SEM Morphology (Figure 5)

**Figure 5:**
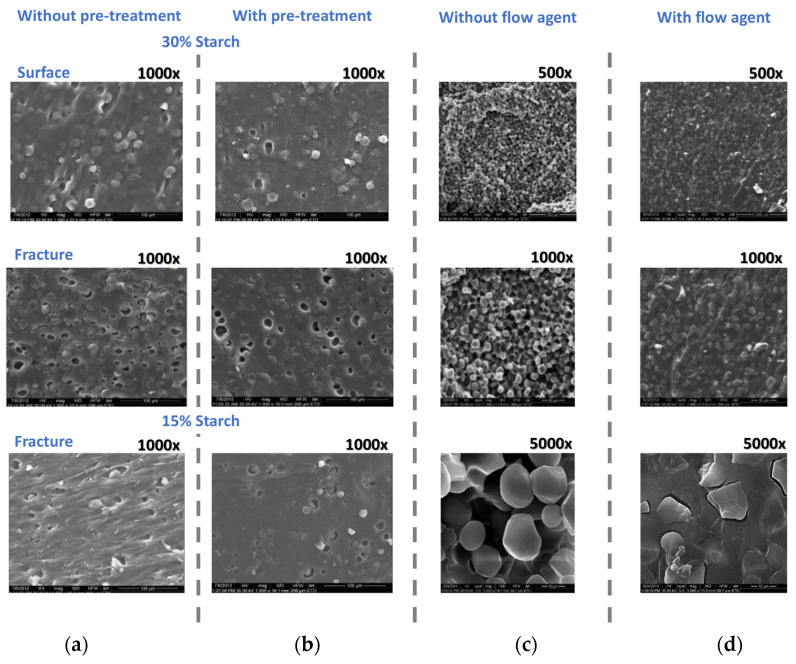
Dependence of the morphologies of the starch–PVA blends on the pre-compounding treatment ((**a**)—without; (**b**)—with) and on the formulation with a flow agent ((**c**)—without; (**d**)—with). 70/30 starch–PVA compound (**a**,**b**); 50/560 (**c**,**d**).

The de-structuring/entanglement compatibilization/structuring sequence described in Section 2.1 reduces the number of morphological defects both on the surface and fractures (Figure 5a–d). As shown, the persistence of the net granule-matrix interfaces, although fewer in number for formulations with a flow agent, indicates that the starch granules could not be completely dispersed into the linear PVA and amylose macromolecules matrix due to the branched amylopectin in the starch (70%) (Figure 5d).

### 3.3. Visco-Elastic Behavior

#### 3.3.1. The Melt Viscoelasticity

##### The Viscous Deformation

The Influence of the Formulation on the Flowing Regime (Shear Stress and Shear Rate)

**Figure 6:**
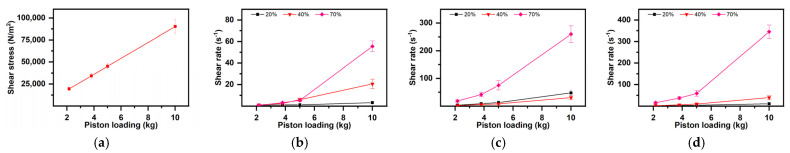
The dependence of the shearing strain and the shearing rate ((**a**)—shear stress; (**b**−**d**)—shear rate) on the starch content and the melt flow conditions (piston loading 2.16 kg−10 kg; temperature: (**b**) 125 °C; (**c**) 135 °C; (**d**) 155 °C).

1. Dependence of shear stress (τ) and shear rate ($\dot{\gamma }$) on the starch content (Figure 6)

The shear stress does not depend on the starch content or the temperature, but it varies with the piston loading (Figure 6a). At small loads of 2.16 kg or 3.8 kg and temperatures up to 135 °C, the shear rate is relatively low and varies significantly with the other flow parameters (Figure 6b–d). At 125 °C, with loads of 5 kg and 10 kg and at a starch content greater than 40%, the shear rate ranges from 55 s^−1^ to 80 s^−1^ (Figure 6b). At 135 °C, its values range from 200 s^−1^ to 250 s^−1^ (Figure 6c), and at 155 °C, it ranges between 300 s^−1^ and 330 s^−1^, indicating that the blends with more than 40% starch that flow at high temperatures and loads develop high shear rates (explained in Section 4).

**Figure 7:**
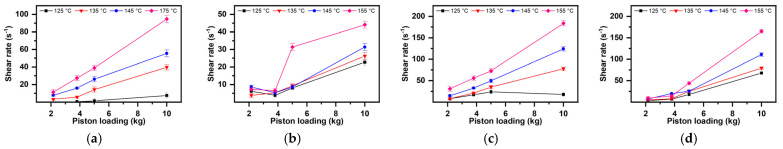
The dependence of the shear rate on the starch amount (20%—(**a**,**c**) and 70%—(**b**,**d**)), and of the plasticizer content (40 [p] (**a**,**b**) or 60 [p] (**c**,**d**)) in 100% starch−PVA blend, the piston load (2.16 kg, 3.8 kg, 5 kg, 10 kg), and temperature regime ((**a**)—125 °C; (**b**)—135 °C; (**c**)—145 °C; (**d**)—155 °C).

2. Dependence of the shear rate on the plasticization degree (Figure 7, Table 1)

The variation of the shear rate in formulation with 1.5 times higher plasticizer amounts ([60] p and [40] p in 100 [p] starch–PVA blends) depends on the starch amount in the blend. At low concentrations of 20% starch, a 2–2.5-fold increase at 155 °C across the entire range of mechanical strains (2.16–10 kg) was registered. Much smaller variations were recorded for blends with 70% starch; when the plasticizer quantity was increased similarly, the shear rate remained approximately constant at low loads of 2.16 kg or 3.8 kg and increased by 1.46–2.33 for higher loads of 5 kg and 10 kg.

**Table 1:** Variation of the shear rate with the formulation (the amount of starch and plasticizer) and with the flow conditions (T = 125–155 °C, load 2.16–10 kg).

Code/Shear rate, s^−1^/Temperature and weight range	$\dot{\gamma }$ $s^{-1},\;155\;{}^{\circ}C\;(\mathrm{Weight}\_f,\;\mathrm{kg})\;-\;\dot{\gamma }$ s^−1^, 155 °C (Weight_i, kg) for 40 [p] plasticizer/Item for 60 [p] plasticizer	
	$\dot{\gamma }$ $s^{-1},\;125\;{}^{\circ}C\;(\mathrm{Weight}\_f,\;\mathrm{kg})\;-\;\dot{\gamma }$ s^−1^, 125 °C (Weight_i, kg) for 40 [p] plasticizer/Item for 60 [p] plasticizer	
	20% starch	70% starch
A. kg	3.8–16 kg	
A1—155 °C, s^−1^	28 ÷ 12/60 ÷ 30	12 ÷ 6/18 ÷ 8
A2—125 °C, s^−1^	2 ÷ 1/18 ÷ 5	3 ÷ 2/4 ÷ 2
A1/A2	14 ÷ 12/3 ÷ 5	4 ÷ 3/4.5 ÷ 4
B. kg	5–3.8 kg	
B1—155 °C, s^−1^	40 ÷ 30/75÷ 55	17 ÷ 12/42 ÷ 18
B2—125 °C, s^−1^	4 ÷ 3/22 ÷ 17	7 ÷ 3/20 ÷ 18
B1/B2	10 ÷ 10/3.4 ÷ 3.24	2.43 ÷ 4/2.1 ÷ 1
C. kg	10–5 kg	
C1—155 °C, s^−1^	93 ÷ 40/180 ÷ 75	40 ÷ 17/160 ÷ 42
C2—125 °C, s^−1^	8 ÷ 4/57÷ 20	22 ÷ 17/60 ÷ 18
C1/C2	11.6 ÷ 10/3.16 ÷ 3.75	1.82 ÷ 1/2.66 ÷ 2/33

**Figure 8:**
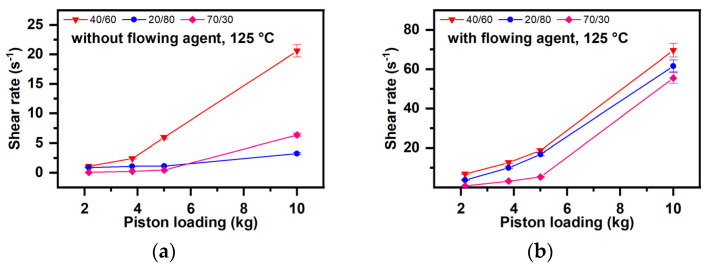
Dependence of the shear rate on the lubrication degree ((**a**)—without flow agent, (**b**)—with flow agent).

3. Dependence of the shear speed on the lubrication degree (Figure 8)

In the range of thermo-mechanical stresses studied, the formulation with the flow agent results in the values of the shear rate increasing by 20–30 times at high stresses of 10 kg.

Influence of the Formulation and the Flow Conditions on the Fluidity

**Figure 9:**
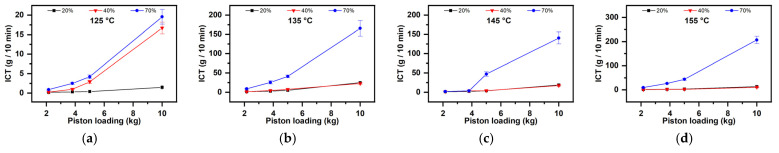
Dependence of the fluidity on the blend starch quantity in the compound (20%—black, 40%—red, 70%—blue; plasticizing level: 60 [p] plasticizer at 100% (Starch–PVOH) blend), the plastometer loading (2.16 kg, 3.8 kg, 5 kg, 10 kg), and the temperature values (**a**)—125 °C; (**b**)—135 °C; (**c**)—145 °C; (**d**)—155 °C.

(i) The dependence on the starch quantity (Figure 9)

At 125 °C, regardless the loading value, the blend with 20% starch exhibits a slightly similar flow behavior (Figure 9a). The fluidity increases approx. 20 times if the load is 3.8 kg or greater (Figure 9a).

**Figure 10:**
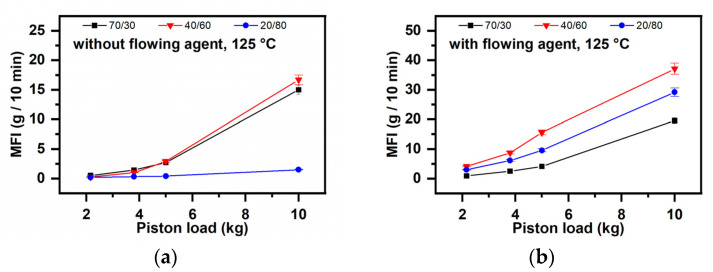
Influence of flow agent on the fluidity of blends with variable starch amounts (starch–PVOH blends with 70% (black), 40% (red), and 20% (blue) starch) (**a**)—without flow agent, (**b**)—with flow agent.

At 135–145 °C, and for 20–40% starch content of the compounds, the fluidity does not change significantly with increasing the plastometer load. Its values can be up to 20 g/10 min at all the studied loads (Figure 9b) and can reach up to 160–180 g/10 min at 155 °C. For the blend with 70% starch, under the same flow conditions (Figure 9c, curve 4), the fluidity can reach up to 200 g/10 min, which can demonstrate an additional degradation process possible at temperatures higher than 145 °C, as explained in Section 3.1.1.

(ii) Dependence on the lubrication degree (Figure 10)

At small loads up to 5 kg and for at all temperatures across all compositions, the lubricant increases fluidity approx. 10 times, especially for the variants with low starch content. At loads greater than 5 kg, the presence of the lubricant increases fluidity by 15–35 times more than for the blend without a flow agent at small loads (2.19 kg, 3.8 kg).

The Influence of Formulation and Flow Conditions on the Melt Flow Resistance (Dynamic Viscosity)

**Figure 11:**
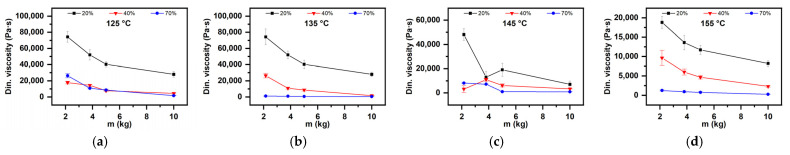
Dependence of the flow resistance on the starch content of the blend (20%—black, 40%—red, 70%—blue), the plastometer loading (2.16 kg, 3.8 kg, 5 kg, 10 kg), and the thermal regime (**a**)—125 °C; (**b**)—135 °C; (**c**)—145 °C; (**d**)—155 °C.

(i) The starch quantity (Figure 11)

**Figure 12:**
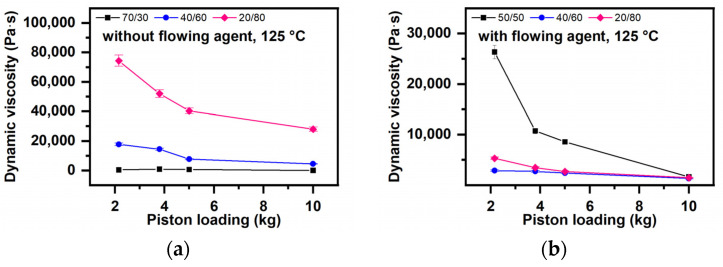
The influence of the flow agent on melt flow resistance of the blend with varying starch content without (**a**) and with (**b**) a flow agent.

For the blend with 20% starch at 125 °C, the melt flow resistance decreases by 44–31% across the entire load range (2.18–10 kg). At 135 °C, the decrease in flow resistance is between 56–80% for flows under small loads of 2.18 kg or 3.8 kg. At 145 °C, the decrease is between 90 and 95%, resulting in slightly rough extrudates, and at 155 °C, the diminishing is ranged as 73–66%, resulting in very rough extrudates.

(ii) Lubrication degree (Figure 12)

Melt flow resistance decreases incredibly with addition of a flow agent, especially for blends with low starch content (under 50%). For example, in a blend with 70% starch that contains a flow agent and flows under 10 kg, the melt flow resistance reaches a maximum of 5000 Pa·s.

##### The Melt Elasticity (Extrudate Swelling) (Figure 13)

**Figure 13:**
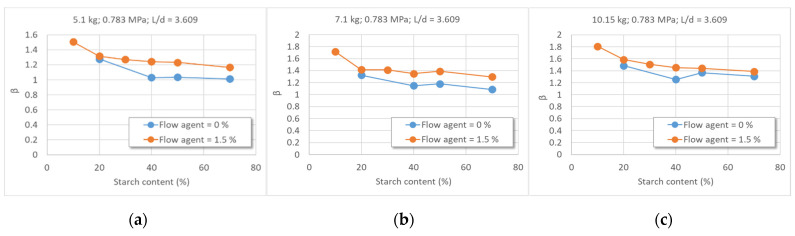

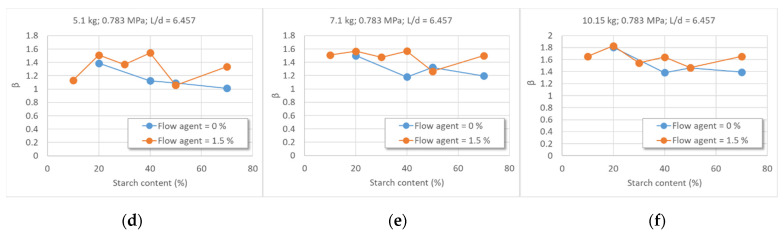
The influence of the flow agent on the extrudate swelling (coefficient β) during melt flow of the blend with varying starch content at T = 125 °C, through various nozzles (L/D of 6.457 and 3.609) and the different loads (G = 5.1 kg; G = 7.1 kg; G = 10.15 kg). (L/D = 6.457, (**a**–**c**); (**a**)—5.1 kg; (**b**)—7.1 kg; (**c**)—10.15 kg; L/D = 3.609 (**d**–**f**), (**d**)—5.1 kg; (**e**)—7.1 kg, (**f**)—10.15 kg).

The melt flow through the nozzle with L/D = 3.609 is accompanied by extrudate swelling, less so for blends with more than 40% starch (Figure 13a–c). The compound with 70% starch that does not contain a flow agent has an extrudate diameter equal to the nozzle diameter (Figure 13a).

If the nozzle is smaller (L/D = 6.457), the melt instability disappears, and swelling decreases with an increase in the starch concentration (Figure 13d–f). For compounds with more than 40% starch without a flow agent, swelling values range from 1–1.2 below 7.1 kg and from 1.2–1.4 under 10.15 kg (Figure 13d). For the same nozzle (L/D = 3.609), the flow agent increases the extrudate swelling for all studied compounds, up to 1.2 below 5.1 kg (Figure 13d), up to 1.4 under 7.1 kg (Figure 13e), and up to 1.4–1.6 below 10.15 kg (Figure 13f). At concentrations higher than 20% starch, the swelling decreases with a steeper slope for formulations without a flow agent and with a smaller slope for those with flow agent (Figure 13a–c). Consequently, the extrudate swelling depends on the flow conditions, the nozzle type. For large nozzle, at high load the swelling increases for almost all formulations with flow agent.

#### 3.3.2. The Viscoelasticity in the Solid State

##### Dynamo-Mechanical Properties (Figure 14)

**Figure 14:**
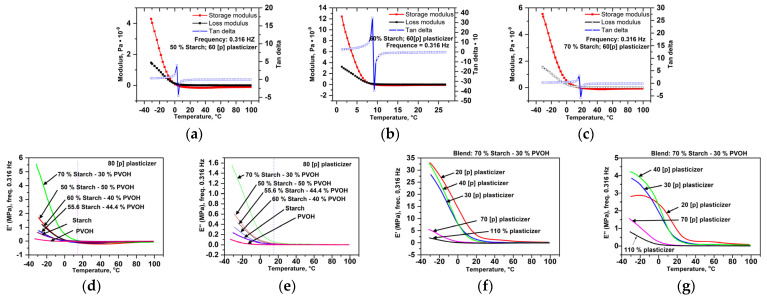
Temperature dependence of elastic (storage modulus) and viscous behavior (loss modulus) and glass transition values based on the starch content ((**a**)—50−70%, (**a**–**e**)) and degree of plasticization ([60] [p] (**a**–**c**); [80] [p] (**d**,**e**); 20 [p]–110 [p] (**f**,**g**)).

Depending on the starch amount and the plasticization degree, for blends with more than 50% starch and 60 [p] plasticizer, the glass transition of starch–PVA compounds decreases from 225 °C (starch) and 84 °C (PVA) to values ranging from −20 °C to 30 °C (Figure 14a–c). At 80 [p] plasticizer and starch content of 55.6% and 70%, the storage modulus at −20 °C ranges between 1 MPa and 5.5 MPa, while the loss modulus ranges from 0.1–1.5 MPa (Figure 14d,e).

According to the values of the storage modulus (elastic deformation), plasticization increases the capacity of compounds with 70% starch to deform elastically by 5–6 times if the amount of plasticizer is 20–40 [p]/100 mixture, but decreases this capacity at higher quantities (Figure 14d, f). The plasticizer accentuates the viscous behavior (as shown by the loss modulus) for the mixture with 70% starch, but only within a certain concentration range [20,21,22,23,24,25,26,27,28,29,30,31,32,33,34,35,36,37,38,39,40] p to 100 [p] blend of starch with PVA. In plasticized PVA–starch compounds (Figure 14d–g), if the blend contains 70% starch, then in the range 0–7.5, the transition from the glassy zone to the highly elastic zone occurs when the storage modulus decreases from 5.5 MPa to 2.25 MPa (Figure 14d–g). At 50% starch in the blend, in the same temperature range, there is a drop in the storage modulus from approx. 4 MPa to 2.15 MPa. The increasing of the plasticizer amount decreases both the storage module (Figure 14f,g) and the loss module.

##### Mechanical Properties

The obtained results show that, in the absence of flow agents and with a starch quantity greater than 50%, the blends are brittle, with low ductility and toughness, showing predominantly viscous breaking. At a plasticization degree of ([40 or 80] p) at 100 % starch–PVA compound, these blends have a breaking resistance of 10 daN/cm^2^–15 daN/cm^2^ and a breaking elongation of 200–300%. If the starch content is less than 50% and the formulation includes a flow additive, the blends are ductile, toughened, and have a medium breaking elongation of of 200% to 480%, indicating essentially viscoelastic behavior.

##### Other Properties: Density: 1.23–1.31 g/cm^3^; Measured Values of Hardness Were Ranged as 55–70 °ShA

**Figure 15:**
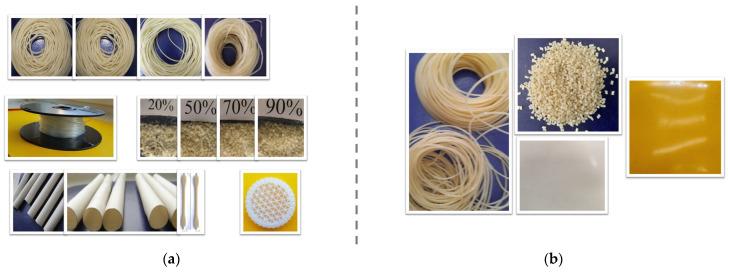
New products obtained by scaling up the selected formulation based on starch [93]: (**a**) initial; (**b**) after 10 years.

Scalable Properties—Thermodynamic Stability (Figure 15)

Starting from the glass transitions of 225 °C for starch and 85 °C for PVA (Figure 1), compounds with glass transitions greater than −20 °C and lower of 30 °C (Figure 14) were realized. The glass transition values depend on formulation, characteristics of the melt compounding devices, and flow conditions. The new compounds have viscoelastic properties in both the melted and solid states, allowing, after proper formulation selection, the transformation into finished products through various techniques such as extrusion, injection, 3D printing. The selected compounds proved good melt processability for finished products by classical (extrusion, injection) or current (3D printing) techniques (Figure 15). The obtained products exhibited excellent thermodynamic stability, maintaining their integrity under ordinary conditions for over 10 years. The selected formulations and working conditions for obtaining compounds with good long-term behavior have been patented or are in process of being patented.

## 4. Discussion

The FTIR study (Section 3.2.1) (ch. 3.2 a) demonstrated that blends with less than 50% starch and those with more than 50% starch, considering the pre-compounding and selected melt compounding conditions, are thermodynamically miscible because the absorption peaks differ from those of the individual components, both in terms of absorption wavelength and intensity. There are two situations where the peaks of the individual components are present but only appear as shoulders in the selected compounds at a shifted wavelength. The amount of starch influences the intensity of the absorption peaks, with the peaks being lower for the compounds with more than 50% starch. This means a slightly reduced kinetic independence of the specific functional groups and, therefore, a higher amorphous content, which is explainable for these blends due to the higher amylopectin content.

These results confirm that, using the described procedure, both starch and PVA were de-structured, and as a consequence of the melt entanglement between starch macromolecules, PVA, and interspersed glycerin macromolecules, new secondary bonds were formed (entanglement compatibilization), making the re-structuring into new compounds with less or more 50% starch content possible. The SEM analysis showed that both the pre-compounding treatment (Figure 5a,b) and the formulation with a flow agent (Figure 5c,d) resulted in a continuous phase appearance of the blends, with few fracture-type boundaries at the border between the branched amylopectin and the matrix represented by the two linear polymers, amylose and PVA.

The “dry-blend” pre-compounding treatment promoted the diffusion of the plasticizer and its absorption into the pores of each solid particle [94]. The proof of the absorption process is that, at the end of the procedure, the mixture of the two solid polymers was not agglomerated and appeared as a powder without liquid content, with free flow of each solid particle (Figure 2). The physical interactions between the plasticizer molecules and the macromolecules of the two polymers, compatibilized through entanglement, decreased the glass transition temperature [95] and contributed to the elimination of the plasticizer leaching.

The new starch–PVA compounds have viscoelastic properties, meaning they combine elastic solid properties (at small values, the deformation is proportional to the applied strain and not depends on the deformation rate) with those of Newtonian fluids (the deformation depends on the deformation rate) [96,97]. The melts of these blends have a viscoelastic character, meaning they deform viscously by dissipating energy [98,99,100] but also accumulate energy in elastic deformation [93,101,102]. Due to viscous deformation processes such as molecular disaggregation, deformation, orientation, and extension of macromolecular chains [98,99,100], polymers can be compatibilized through melt entanglement. The shear strain, defined as the ratio between the force F applied tangentially to the flow direction and the flow area [103,104], does not depend on the starch quantity, temperature, or load (Figure 6a) (Section 3.3.1) (Section 3.3, a, a1, A, i). The shear rate, representing the rate gradient in the transverse direction when the flow takes place between two parallel planes, indicates the rate of change of one material layer relative to another, serving as an implicit measure of homogenization capacity [105]. The shear rate depends on the blend formulation and on flowing conditions (temperature and load). If homogenization takes place at high temperatures, high loads, and the blends have more than 50% starch, a high degree of plasticization, and a flow agent (Figure 6a,b), the magnitude of the developed shear rates (Figure 4b–d, Table S1) facilitates easier homogenization. Fluidity, analyzed based on MFI values, represents the material amount that flows through a nozzle of the selected diameter over 10 min [106,107,108]. High and very high fluidity values can also occur due to parallel degradation processes involving starch and PVA. Fluidity increases with starch content (Figure 9a–d), formulation with lubricant (Figure 10a,b), and increasing temperature and load during melt compounding (Figure 9a–d). If degradation is avoided, these possibilities increase the homogenization degree. Dynamic viscosity or melt viscosity (Melt flow Resistance) represents the internal friction in a melt when one fluid layer moves relative to another (Newton’s law of friction) [109]. According to Figure 11 and Figure 12, the melt flow resistance, or the internal friction occurring in the melt when one layer of fluid moves relative to the other [110,111,112,113], decreases with the increase in the starch amount due to the presence of a flow agent, as well as with the increase in temperature and load. The flow resistance controls the degradability of macromolecules in the melted state. Consequently, the melt viscous deformation that conditions the achievement of a high miscibility degree and non-degraded polymer blends is controlled by the characteristics of the compounding device, the shear strain values, and the operating conditions through values of shear rates, fluidity, and melt flow resistance. The plastic deformation must be controlled to enable the de-structuring of both polymers and the entanglement of the resulting macromolecules, allowing new attractions between starch and PVA, making it possible to re-structure into new compounds with advanced macromolecular miscibility. According to [114], shear rates of 0.5 s^−1^–3000 s^−1^ are needed for achieving good entanglement during compounding, while shear rates of 1 s^−1^–103 s^−1^ are required for extrusions—filaments/films/fibers, and 102 s^−1^–105 s^−1^ for injection.

Based on these results, considering the characteristics of the devices in which the compounding and melt processing into the final product will take place, formulas and thermo-mechanical working conditions are chosen for each desired situation. This ensures that the de-structuring, melt entanglement of free-flowing macromolecules, and re-structuring processes lead to miscible, non-degraded compounds with desired functional properties, allowing for good processability into the finished product using the appropriate technique.

The melt elastic deformation (extrudate swelling and the Barus effect [115]) occurs during filaments formation for granulation and the production of the finished product as it flows through a nozzle during extrusion and/or injection [116]. In the nozzle, macromolecules stretch, reducing their volume, and upon exiting, they tend to return to their original balk state. The difference between the initial deformation and the recovered state (elastic deformation) is called viscous deformation [117]. Uneven extrudate swelling at the nozzle exit will lead to products with geometries different from that of the nozzle [117,118,119]. Residual stress from unrecovered viscous deformation diminishes the quality of plastic parts, causing subsequent deformations in the solid state, especially in injected products exposed to heat [120]. This stress can result in contraction, cracks, and fractures, negatively affecting the breaking and fatigue behavior. In injection technology, post-molding conditions are important, as the relaxation of macromolecules depends on the mold temperature [121,122]. In blow extrusion, the stretching and cooling rates directly affect the residual stress and the balloon properties [123,124], as well as the quality of the products like bags made from the extruded blow films [125]. In 3D printing, extrudate swelling and the residual stress may occur during both filament production and the 3D printing phase. The release of residual stress through physical or thermal aging leads to the delamination of 3D printed layers, resulting in reduced resistance and longevity [126]. Data from Figure 13 show that, depending on the formulation, flow conditions, and nozzle geometry, the diameter of the extrudate from the new starch–PVA compounds can be equal to or up to 60–80% greater than the nozzle diameter. Therefore, the formulation and melt compounding conditions will thus be selected to ensure that extrudate swelling does not compromise the quality of the targeted finished product.

The moduli–temperature dependences, which depict the regions of viscoelastic behavior in the solid state, are described by two plateau areas: one for the glassy state and another for the highly elastic state. Between these is a sharp drop in the moduli at very small temperature increases, which marks the glass transition [127,128]. The tensile storage modulus E’ (or shear G’) measures the stored energy without breaking, describing the elastic behavior. The tensile losses modulus E” (or shear G”) measures the dissipated energy as heat, indicating the viscous behavior. Tan delta or the damping factor (dissipation factor) represents the ratio between the viscous and elastic response of the studied mixtures [129]. The vitreous transition increases with the starch amount (Figure 14a–c), most likely due to the limitation of macromolecular displacements resulting from the amylopectin cluster structure (Figure 14d,f). The storage modulus is higher for the compounds with more starch, most likely due to the matrix’s capacity to absorb deformations and the amylopectin branches (Figure 14e,g). The viscous behavior, reflected by the loss modulus (Figure 14) of the compounds with more than 40% starch, may result from insufficient plasticizer homogenization into the polymer matrix and the higher amylopectin content, which struggles to return to its initial state after deformation due to its breached structure. The increasing of the plasticizer amount decreases the elastic deformation capacity (Figure 14 f) because the macromolecules, having higher mobility, cannot easily return to their state before the deformation. These macromolecules, being more flexibility due to the amount of plasticizer, form structures with fewer network defects, resulting in compounds with less viscous deformability (Figure 14g).

Understanding the viscoelastic properties in the solid state is essential for selecting melt processing techniques for new compounds into finished products and ensuring their functional properties. For example, the formulations with high energy storage capacity will be selected for blow molding films or foils and extrusion filaments for 3D printing. Blown compositions must have high stretching and elastic deformation capacities to ensure that the bags made from these films exhibit good breaking behavior. The formulations for 3D printing must not swell at the nozzle exit; they must deform plastically during filament formation with the proper diameter for printing and maintain their dimensions. These compositions must have a stable melt flow; otherwise, the deposition of successive layers may not be uniform.

The compounds with a high ability to dissipate deformation energy will be selected for thermoforming [130], when the shape of the product is obtained as a result of plastic deformation. t. These products must maintain their shape throughout their entire lifetime.

To ensure good performance, the formulation selection must consider all the functional properties (physical, mechanical, etc.) according to the requirements of the processing technology. The constant diameter and ovality of nearly 1 of the 3D filaments demonstrated that the selected compositions’ extrusion was not accompanied by swelling at the nozzle. The obtained results showed that the blends with less than 50% starch, which deform elastically in solid state, are suitable for melt processing into 3D films or filaments, as observed [131]. Special care was given to the flow conditions to avoid the exudate swelling. The blends with more than 50% starch, which withstand high plastic deformations, were processed into finished product through injection, thermoforming [130], etc. The selected formulations and working conditions are the subjects of several patents and patent applications.

An interesting result is the long durability of the selected new compounds. This durability is likely due to good dispersion of minority components in the basic polymer matrix and the high thermodynamic miscibility achieved through pre-compounding treatment and the melt compounding at proper shear rates melt fluidities, and optimal melt flow resistance to achieve homogenization without degradation. The applied procedures facilitated the de-structuring of the two polymers, starch and PVA, macromolecules entanglement, and re-structuring into new, thermodynamically stable compounds with scalable properties. The thermodynamic stability will be quantitatively described through comparative FTIR, DSC, etc., analyzes, initially and after 10 years, with results presented in a future article. These results are directly related to the approach of improving the miscibility for increasing the lifetime of compounds containing renewable-based polymers. After further investigation of the thermodynamic stability over time, it may be possible to expand the use of starch in medium-life compositions, as opposed to current application in short-life items.

## 5. Conclusions

1. The aim of the article was to design and develop new starch-based compounds that are melt-processable into finished product using scalable, classic (extrusion, injection, thermoforming) or 3D printing techniques. In order to achieve this goal, the powder of starch and PVA was blended with a liquid plasticizer in a pre-compounding sequence carried out under selected thermal and mechanical conditions. The melt compounding of these solid blends occurred under conditions that allowed the de-structuring of the two polymers, the melt entanglement of the resulting macromolecules, and the re-structuring of the new, non-degraded compounds. These compounds exhibited high thermodynamically miscibility, good functional properties, and scalable properties. These compounds were selected based on both their molten and solid-state viscoelastic properties and their functional properties. The melt processing of the selected compounds using various techniques demonstrated good scalable properties and a lifetime exceeding 10 years.
2. Starting from starch with glass transition at 125 °C and PVA with T_g_ at 85 °C, following the described working sequence, new starch–PVA compounds with glass transitions between −20 °C and 50 °C were made, showing optimal melt processability using both classic (extrusion, injection) and 3D printing methods. The homogenization degree was controlled by the extent of melt viscous deformations, which depends on shear rates, fluidity, and the melt resistance to flow to prevent polymer degradation. The extrudates swelling was eliminated by managing the melt’s elastic deformability. All these melt rheological properties were influenced by formulation, flow conditions, characteristics of the compounding device, and the dimensional characteristics of the nozzle. Formulations and compounding conditions were selected to allow processing at temperatures below 140 °C.
3. The thermodynamic stability will be quantitatively described through comparative FTIR, DSC, etc., analyses, initially and after 10 years, in a future article. Further examination of the time-based thermodynamic stability features of these compounds may pave the way for using starch in medium-life compositions, beyond its current application in short-life products.
4. The incomplete melting and rough surfaces were avoided by using only dried polymers before melt compounding, working only with polymers of similar and nearly equal particle diameter, using formulations with flow agents, and controlling deformability in the melted state.
5. The FTIR study demonstrated the advanced miscibility of the compounds made using the specified sequence.
6. The viscoelastic behavior in the solid state allowed the selection of the formulations with viscous deformability to ensure optimal stretchability for extrusion-blowing and 3D printing processes. The formulations with viscous deformability were selected to ensure good behavior in injection molding and thermoforming processes.

## Data Availability

The original contributions presented in the study are included in the article/Supplementary Materials, further inquiries can be directed to the corresponding author.

## Supplementary Materials

The following supporting information can be downloaded at: https://www.mdpi.com/article/10.3390/polym16213063/s1, Table S1: Comparative analysis of the FTIR spectra of the blends with 20% and 70% starch compared with those of the individual components.
