# Supplementary material for: De- and Re-Structuring of Starch to Control the Melt and Solid State Visco-Elasticity as Method for Getting New Multi Component Compounds with Scalable Properties
Source: Polymers (Basel). 2024 Oct 30;16(21):3063. doi: 10.3390/polym16213063 (PMC11548643; doi:10.3390/polym16213063)
Supplement: Supplementary file 1 [file polymers-16-03063-s001.zip › polymers-3270977-supplementary.pdf]

Table S1. Comparative analysis of the FTIR spectra of the blends with 20% and 70% starch compared with those of the individual components.

**Spectral range, cm^{-1}**	**Wave number, cm^{-1}/ Intensity, a.u**				**Comments**
	Compound	Starch	PVA	Plast.	
**-3000<br>(0-H stretch)**	a)20 % S<br>M:3340 / 0.25<br>b)70 % S<br>M:3340 /0.20	3273 / 0.15	M:3286 / 0.051	M:3274 / 0.32	From 3 P - resulted 1, without Shd. Near S/PVA/G: shifting with 67 cm^{-1} /54 cm^{-1} / 66 cm^{-1} .<br>HP: (a) 20 % S: ↑ 66 % / ↑ 80 % /↓ 22 %<br>(b) 70 % S: ↑ 46 %, ↑ 77%, ↓32 %
**3000 -2800<br>(C-H and)-H stretch**	a)20 % S<br>M:2916/0.125<br>b)70 % S<br>M:2940 / 0.09	2920 / 0.08	M;2935/ 0.043<br>M:2909 / 0.04	0.18<br>0.18	From 5 P- resulted 1 P at 2916 cm^{-1} for (a) and 2940 cm^{-1} for (b) Shd: (a) la 2851 cm^{-1} (b) la 2912 cm^{-1} . P for a) shifted with 7 cm^{-1} near PVA. P for b) shifted with 20 cm^{-1} near 2920 cm^{-1} from S and with 5 cm^{-1} near 2935 cm^{-1} from PVA. I ↑ near I from S, PVA, P for a) cu 36 %; 34 %; 44 %; for b) cu 12 %; 63 %; 19 %.
**1750 -1500<br>(C=O and C-C stretch)**	a)20 % S<br>M: 1624/0.245<br>Shd:1713 si 1663<br>b)70 % S<br>M: 1624/0.21<br>U:1713 si1663	1637 / 0.03<br><br><br>Idem	M:1731 / 0.05<br>M: 1569/0.01<br><br>Idem	M:1653 / 0.04<br><br><br>Idem	From 4 P - resulted 1 P with Shd. a): M -new, Shd:1713 shifted with 60 cm^{-1} near 1569 cm^{-1} din P; 1663cm^{-1} with 26 cm^{-1} near S from 1637 cm^{-1}. 1731 cm^{-1} doesn't appear. I: Near S/PVA/G: ↑83 % / 77 % - 98 %/82 %. b)<br>b) Near S/PVA/G smaller with ↓83 %/↓77 % - 96 % /↓ 82 %
**1500 – 1300<br>(C-C stretch, C-H rock)**	a)20 % S<br>M:1444 / 0.15<br>Shd:1375 si 1332<br>b)70 % S<br>M:1452<br>Shd: 1375 s1335	M: 1337 / 0.08<br>Shd.: 1415<br><br>Idem	M1:1432 / 0.04<br>M2:1372 /0.042<br>Shd:1329 /<br>Idem	Max: 1416/0.1<br>Shd1:1455<br>Shd2:1329<br><br>Idem	From 5 P with 5 Shd -resulted 1 with 2 Shd:(a) P 1444 cm^{-1} can be the SHd from G from 1445 cm^{-1}. 1375 cm^{-1} -shifted with 37 cm^{-1} near M from S at 1337 cm^{-1}. The Shd from 1375 cm^{-1} from PVA at 1372 cm^{-1} shifted with 2 cm^{-1} a. Shd from 1332 cm^{-1} can be the shd from 1329 cm^{-1} from PVA and/or P shiefted with 3 cm^{-1}. I near S/PVA/P: ↑47 %/↑73 %-21 % / ↑33 % (b) P can be the Shd from G at 1445 cm^{-1} shiefted with 8 cm^{-1}. I: ↓ 20 %/↓50 %/↓67 %. Shd idem with (a).
**1300 – 1180<br>(C-O stretch)**	a)20 % S<br>M:1250 /0.15<br>b)70 % S	1240 /0.05	M:1240/0.075	M:1213/0.05	Form 3 P -resulted 1 shifted with 10 cm^{-1} fata de S and PVA and a T 37 cm^{-1}: I near S/PVA/G: ↑ 67 %/50 %/67 %.<br>Idem; I: near S/PVA/G: ↑ 53 %/ 45 %/ 59
**1180 -950<br>(C-O strtche, O-H bend**	a)20 % S<br>M:1050 / 0.285<br>b)70 % S<br>M:1024 /0.23<br>Shd: 1077, 1044	M: 1149 / 0.1<br>Shd:1077/0.15<br>Shd:955/0.37<br>Idem	M:1088/0.08<br>M:1024/0.07<br><br>Idem	M:1109/0.18<br>Shd1:1035/0.45<br>Shd2:994/0.18<br>Idem	a) From 8 P-resulted 1 which can be shiftat with 55 cm^{-1} near 955 cm^{-1} from S; With 15 cm^{-1} and 64 cm^{-1} near P from 1035cm^{-1} si 944 cm^{-1} from PVA and G. Do not apper: 1149 cm^{-1} and 1077 cm^{-1} from S; 2 P from PVA and 1109 cm^{-1} from G<br>b) From 8 P- resulted 1 with absorbtion at 1024 cm^{-1} as in PVA. The Shd from1077 cm^{-1} identically as in S. 5 absorptions disappear. Aare higher than the A from individual components with the following exceptions when they are lower by 60% compared to 955 cm^{-1} from S and by 60% compared to 994 cm^{-1} from G.
**950 – 900<br>(O-H bend)**	a)20 % S<br>M :922 / 0.03<br>b)70 % S	M:930/0.08	M:945/0.02	M:994/0.18<br>M:924/0.15	From 4 P -resulted 1 without Shd. All the other P of individual components disappear.<br>Idem
**900 – 700<br>(C-H oop)**	a)20 % S<br>851/0.75<br>b)70 % S<br>852 /0.8	M:859/0.06<br>M:762/0.062	M:845/0.035	M:924/0.085<br>M:852/0.05	From 5 P-resulted shifted with 6 cm^{-1} near 845 cm^{-1} from PVA; the same with those from G from 852 cm^{-1}. 3 P do nor apppear. I: 90 % ↑ as individual components<br>Idem a)
